# Rapid and discriminatory diagnosis of scrapie and BSE in retro-pharyngeal lymph nodes of sheep

**DOI:** 10.1186/1746-6148-2-19

**Published:** 2006-06-09

**Authors:** Jan PM Langeveld, Jorg G Jacobs, Jo HF Erkens, Alex Bossers, Fred G van Zijderveld, Lucien JM van Keulen

**Affiliations:** 1 Central Institute for Animal Disease Control Lelystad (CIDC-Lelystad), PO Box 2004, 8203 AA, Lelystad, The Netherlands

## Abstract

**Background:**

Diagnosis based on prion detection in lymph nodes of sheep and goats can improve active surveillance for scrapie and, if it were circulating, for bovine spongiform encephalopathy (BSE). With sizes that allow repetitive testing and a location that is easily accessible at slaughter, retropharyngeal lymph nodes (RLN) are considered suitable organs for testing. Western blotting (WB) of brain homogenates is, in principle, a technique well suited to both detect and discriminate between scrapie and BSE. In this report, WB is developed for rapid diagnosis in RLN and to study biochemical characteristics of PrP^res^.

**Results:**

Optimal PrP^res ^detection in RLN by WB was achieved by proper tissue processing, antibody choice and inclusion of a step for PrP^res^concentration. The analyses were performed on three different sheep sources. Firstly, in a study with preclinical scrapie cases, WB of RLN from infected sheep of VRQ/VRQ genotype – VRQ represents, respectively, polymorphic PrP amino acids 136, 154, and 171 – allowed a diagnosis 14 mo earlier compared to WB of brain stem. Secondly, samples collected from sheep with confirmed scrapie in the course of passive and active surveillance programmes in the period 2002–2003 yielded positive results depending on genotype: all sheep with genotypes ARH/VRQ, VRQ/VRQ, and ARQ/VRQ scored positive for PrP^res^, but ARQ/ARQ and ARR/VRQ were not all positive. Thirdly, in an experimental BSE study, detection of PrP^res ^in all 11 ARQ/ARQ sheep, including 7 preclinical cases, was possible. In all instances, WB and IHC were almost as sensitive. Moreover, BSE infection could be discriminated from scrapie infection by faster electrophoretic migration of the PrP^res ^bands. Using dual antibody staining with selected monoclonal antibodies like 12B2 and L42, these differences in migration could be employed for an unequivocal differentiation between BSE and scrapie. With respect to glycosylation of PrP^res^, BSE cases exhibited a greater diglycosylated fraction than scrapie cases. Furthermore, a slight time dependent increase of diglycosylated PrP^res ^was noted between individual sheep, which was remarkable in that it occurred in both scrapie and BSE study.

**Conclusion:**

The present data indicate that, used in conjunction with testing in brain, WB of RLN can be a sensitive tool for improving surveillance of scrapie and BSE, allowing early detection of BSE and scrapie and thereby ensuring safer sheep and goat products.

## Background

Transmissible spongiform encephalopathies (TSEs) or prion diseases are fatal diseases of the central nervous system, characterized by long incubation periods before neurological symptoms appear. TSEs were initially described in sheep as a transmissible disease named scrapie, and in man, as Creutzfeldt-Jakob disease (CJD), a sporadic disease with no detectable link between cases. The diseases can be diagnosed by vacuolation of neurons in the brain and appearance of a structurally altered form of the host encoded prion protein (PrP), termed PrP^Sc^. TSEs represent a unique class of infectious diseases in which PrP^Sc ^is considered to be the transmissible agent [1-3].

TSEs have attracted much attention since bovine spongiform encephalopthy (BSE) was first described in the 1980s [4]. The more or less accepted explanation for the appearance of this condition was the capacity of the scrapie-like agent to spread to cattle fed with insufficiently heated remains of ruminants [5]. In the 1990s a new form of CJD was observed affecting mainly young people [6]. This so-called variant CJD is considered to be caused by the BSE agent [7] and to have spread by the consumption of infected food containing residual bovine nervous tissues, although other means of transmission, such as the use of ruminant-derived substances in medical products, can not be ruled out. Furthermore, man-to-man transmissions might have occurred subsequently by blood transfusions. BSE is now largely under control, but two recent reports describing goats with BSE have strengthened the fear that the BSE agent could again become widespread in some food animals before being diagnosed [8-10]. In order to prevent this from happening, the TSEs from livestock must be eradicated. For this purpose a number of programmes are underway.

For eradication of ruminant TSEs in Europe, where BSE cases have occurred in nearly all member states, control programmes have been implemented (EU legislation 999/2001 and additions), e.g., the removal of risk materials, active monitoring of slaughtered cattle, sheep and goats, stamping out of scrapie, and genetic breeding of sheep towards scrapie and BSE resistant PrPgenotypes. Active monitoring is possible using brain tissue taken late in the incubation phase. Early diagnosis of TSEs can assist in monitoring programs and has potential in sheep and goats where lymphoreticular tissues generally accumulate infectivity and aberrant PrP [11-14]. Indeed, until now, tonsil and other lymphoid tissues have been targets for preclinical scrapie diagnosis in sheep, making reliable diagnosis possible in biopsies early during incubation [15-17]. However, for the diagnosis to be as early as possible, *i.e*. in the preclinical stage, diagnostic methods more rapid than the time consuming microscopic and bioassay techniques currently employed are required. Such a diagnosis should also preferably allow discrimination between TSE strains like scrapie and BSE, as is described for Western blotting (WB) methods applied to brain [18-22].

A very reliable indicator of disease is the presence of abnormal forms (PrP^Sc^) of prion protein. It is recognized by immunohistochemistry (IHC) as accumulations of PrP, or as a partially protease-resistant protein (PrP^res^). By WB of PrP^res ^using PrP-specific antibodies, a typical triplet banding pattern appears, representative of diglycosylated, monoglycosylated and aglycosyl PrP^res ^moieties, migrating with apparent molecular masses between 16 and 30 kDa. Using staining with two antibodies specific for different PrP-sites, WB can even discriminate between scrapie and experimental BSE infection in sheep brain due to differential cleavage of PrP^res ^by proteinase K (PK) [21,22].

In this study we investigated the applicability of PrP^res ^WB to retropharyngeal lymph nodes (RLN) since these organs are easily accessible at slaughter and are one of the best targets for diagnosis in the lymphoid system [13,17,23]. This was performed on three groups of sheep: firstly, sheep from an early and late scrapie pathogenesis study with mainly genotype VRQ/VRQ (only codons 136, 154 and 171 of PrP are indicated in single letter amino acid code); secondly, ARQ/ARQ sheep with orally induced BSE; and thirdly, sheep diagnosed during active and passive surveillance in the years 2002–2003. The latter group represented six different PrP genotypes.

## Results

### Antibody choice and fine epitope specificity

**Figure 1:**
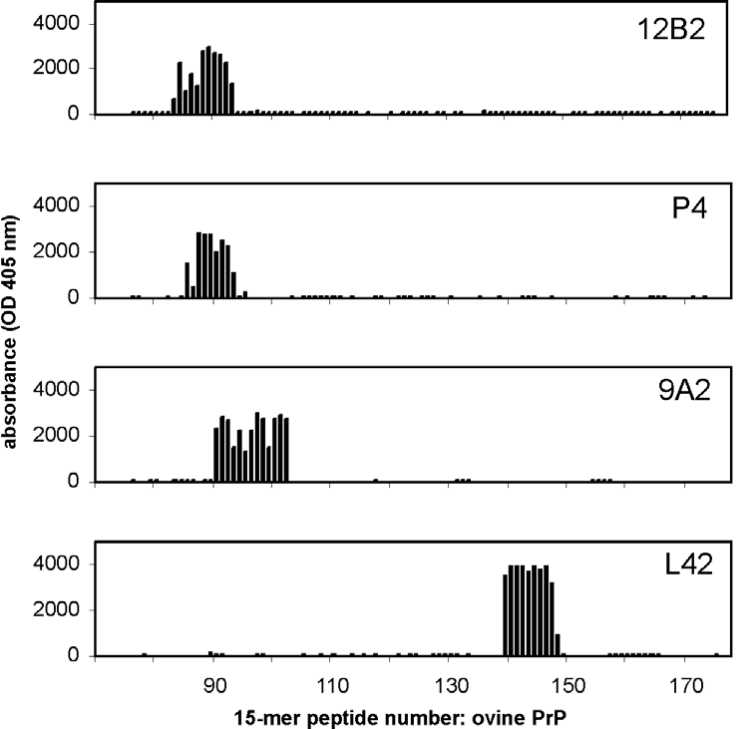
**Mapping of antigenic sites in the ovine PrP sequence for 4 monoclonal antibodies by Pepscan analysis**. For each antibody indicated, results of analyses are presented for peptides 76–175. Antigenic peptides for the respective antibodies 12B2, P4, 9A2, and L42 are: 83–93, 85–93, 90–102 and 139–148. See Table 1 for deduced core of the epitopes.

In lymphoid tissues PrP^res ^concentrations have been reported to be 20 times lower than in brain [24]. Therefore, monoclonal antibodies (mAbs) with superior affinity for ovine PrP in Western blot (WB) were sought. Ten mAbs (SAF84, SAF70, P4, L42, 8G8, 34C9 6H4, 94B4, 12B2 and 9A2) with specificity for ovine PrP were compared on WB strips by varying two parameters: mAb concentration and amounts of ovine scrapie brain digest (see Methods section). As a result, antibodies 12B2, P4, 9A2, and L42 were selected as the most reactive. The epitope specificities of these four mAbs were determined by Pepscan analysis on solid phase synthetic ovine PrP peptides (Figure 1). Synthetic peptides carrying these epitope sequences were also used in solution to investigate by ELISA their capacity to block binding to recombinant ovine PrP. Indeed, as can be expected for antibodies that are elicited with peptides, all four of these could interfere effectively with antibody binding to PrP, confirming the significance of the mapped specificities (Table 1).

### Tissue treatment and choice of retro-pharyngeal lymph node

A practical means of homogenisation was chosen for tonsillar and medial retropharyngeal lymph node (RLN) tissue by using disposible homogenisers available for mass screening purposes. Prypcon homogenisers were preferred since these consistently yielded satisfactory suspensions while other probes caused formation of gel in the homogenate.

**Figure 2:**
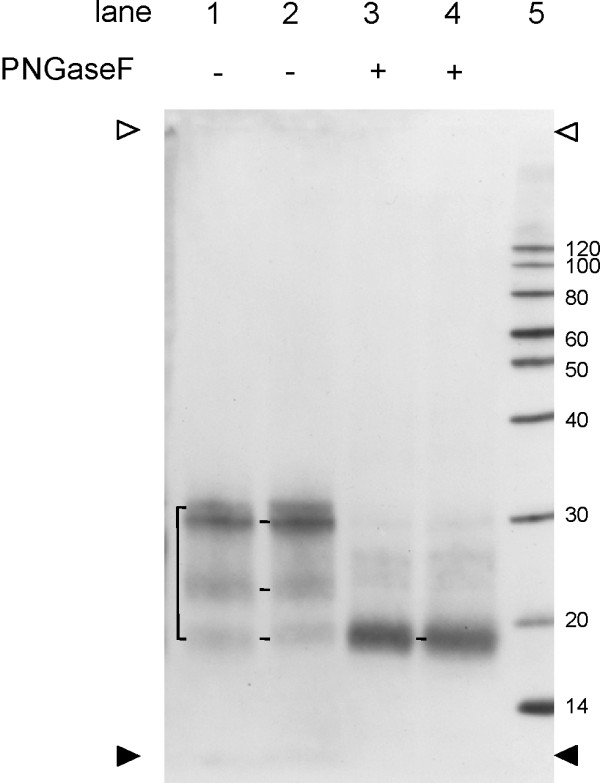
**Western blotting of homogenates from scrapie positive tonsil and retropharyngeal lymph node**. Tonsillar (lanes 1, 3) and medial retropharyngeal lymph node (lanes 2, 4) material digested with proteinase K was analyzed by WB and developed with monoclonal antibody P4 (0.2 μg/ml). Enzymatic treatment with PNGaseF is indicated. Lanes 1–4: Applied amounts 450 μg TE/lane. Lane 5: molecular weight markers. Position of markers are in kDa. Other symbols: open arrowhead for top of gel, bold arrowhead for front of bromphenol blue, bracket for migration position of PrP^res ^triplet, small lines for the three individual PrP^res ^polypeptides in the triplet as discussed in the Results section. Position of markers is in kDa. Band at 14 kDa represents lysozyme from SeeBlue Mark12 which is bound by anti-mouse alkaline phosphatase conjugate.

Homogenates of tonsils and RLN from scrapie-infected sheep were examined after enzymatic hydrolysis with proteinase K (PK) and/or deglycosylation with PNGaseF (Figure 2). Digestion with PK resulted in the typical triplet of diglycosylated, monoglycosylated and aglycosyl PrP^res^. Subsequent removal of carbohydrate moieties by PNGaseF led further to a prominent single deglycosylated PrP^res ^band. There were no major differences in PrP immunostaining between tonsils and RLNs.

In further studies, RLN tissue was used as the preferred tissue for evaluating lymphoid tissues, since in contrast to tonsillar tissue, it lead to reproducible scrapie diagnosis and organ size was large enough for repetitive analysis. This better reproducibility is likely due to a more homogeneous distribution of PrP^Sc ^in RLN compared to tonsils. Also, at slaughter, RLNs are more easily accessible in the decapitated head than are tonsillar tissues.

### Diagnosis of scrapie in sheep during the incubation phase

We wished to know first, whether WB could detect scrapie in the preclinical phase. As reported previously, VRQ/VRQ sheep from our scrapie-infected flock are IHC positive in tonsils and RLN at two months of age [25,26]. RLN samples of 18 such sheep, as well as of 6 scrapie negative sheep (4 VRQ/ARR and 2 ARR/ARR genotypes), were examined by WB. From the age of three months, WB yielded results identical to IHC (Table 2). In 2-month old cases, WB was negative, whereas IHC could detect PrP only in fewer than 25% of the follicles. From the age of four months onwards, all VRQ/VRQ sheep were positive in both IHC and WB. A 20-fold concentration step allowing application of 20 mg tissue equivalents (TE) per lane instead of 1 mg yielded a proportionally stronger signal in the positive cases, but did not change the diagnostic outcome (data not shown). The RLN samples from the 6 scrapie negative sheep remained WB negative.

When tested by WB, brain tissue from the VRQ/VRQ animals scored positive from 17 mo of age onwards, which was some 7 mo later than by IHC. Compared to unconcentrated samples, application of 20 mg TE after a preceding concentration did not lead to earlier detection of PrP^res^. By WB, scrapie diagnosis using RLN from these sheep was already possible at 3 mo of age, while a positive diagnosis with brain was obtained only from 17 mo of age, thus illustrating that WB on lymphoid tissue can lead to a scrapie diagnosis at a very early stage of infection.

### Diagnosis in animals in preclinical and clinical phase of experimental BSE

RLNs from sheep with experimental BSE, in a previous [27] and ongoing study, were examined by WB. All 11 infected animals of this study were positive by WB, but only after concentration to 10–20 mg TE/lane. (Table 3; 4 clinical, 7 preclinical cases). If no concentration treatment was included and mAb L42, the best antibody for BSE, was used, 6 out of 7 animals in the preclinical stage, and 2 out 4 in the clinical stage, could be diagnosed. Thus, in the case of BSE, concentration improves the diagnostic sensitivity. Surprisingly while all animals were positive by WB, one with the shortest duration of infection (6 mpi) was negative by IHC.

**Figure 3:**
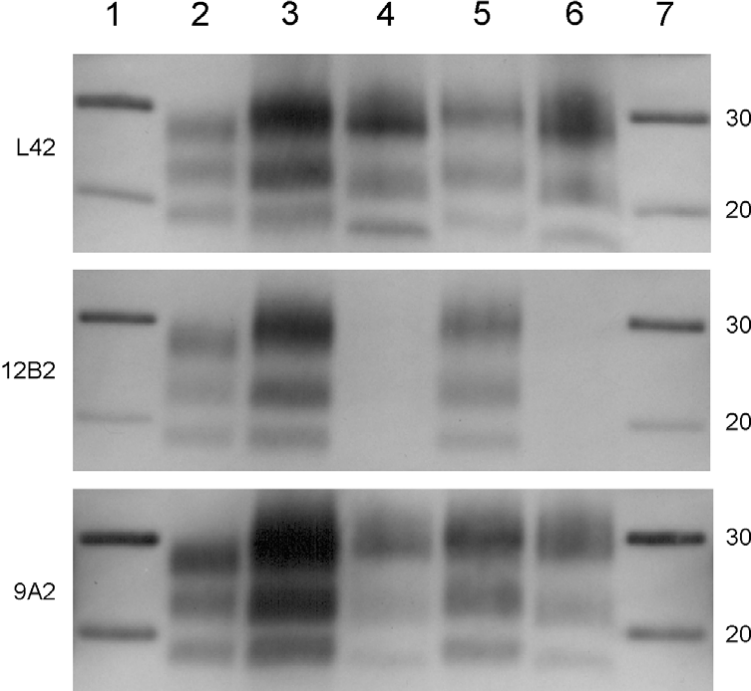
**Typical PrP^res^profiles in scrapie and BSE infected retropharyngeal lymph nodes using different antibodies**. Type of specimen applied: lanes 1 and 7 molecular weight markers in kDa; lane 2 brain stem homogenate from ARQ/VRQ sheep with scrapie; lane 3, RLN from VRQ/VRQ scrapie sheep at age 26 m; lane 4, RLN from BSE-infected ARQ/ARQ sheep in preclinical stage of disease; lane 5, RLN from ARQ/ARQ sheep with subclinical (brain positive, no clinical signs) stage of scrapie; lane 6, RLN from BSE-infected ARQ/ARQ sheep in clinical stage of disease. Amounts of tissue applied: lane 2, 200 μg TE, lanes 3–6, 10 mg TE. Antibody concentrations used: L42 and 12B2, 0.2 μg/ml; 9A2, 2 μg/ml.

Antibodies showed different affinities for PrP^res ^in BSE- and scrapie-infected sheep (Figure 3). PrP-core specific antibodies L42 and 9A2 recognized PrP^res ^in BSE samples, with the former being the best antibody. PK-cleavage site specific antibodies 12B2 (and P4) hardly bound to PrP^res ^from BSEinfected samples. Remarkably, 9A2 bound less strongly to PrP^res ^in BSE samples than L42, contrary to findings with scrapie-infected sheep. It seems, thus, that the 9A2 epitope in BSE samples could be more prone to PK cleavage than that in scrapie. Variable cleavage of PrP^Sc ^is a recognized phenomenon, but the extent or pattern of these cleavages could well depend on agent-specific PrP-conformational properties, which *in situ *might result in differences in antibody binding between potentially different TSE strains [22,27-31]. These results also emphasize that for discrimination between scrapie and BSE in dual antibody staining, the use of core antibody L42 is preferable to 9A2, especially in weakly positive samples.

### Diagnosis of scrapie in field cases from surveillance and with mostly unknown ages

RLNs from sheep with various PrP genotypes and from different sources, were examined by WB and IHC. In general, again, there was a good correlation between the two methods, since divergent results occurred only for some weak IHC-positive cases (Table 4; and *for individual data see Table in *"additional file 1"). Thus, 51 out of 61 brain-positive sheep were diagnosed to be scrapie positive with RLN by both methods and two others were only positive by IHC. Within the different genotypes, WB of RLN scored 5 out of 7 positive in the ARQ/ARQ group, (while IHC scored one more, 6/7), and 1 out 9 (IHC 2/9) in the ARR/VRQ group. For the other genotypes (VRQ/VRQ, ARQ/VRQ, ARH/VRQ, ARQ/ARR), the two tests corresponded fully with each other. In brain-negative sheep, 1 out 7 appeared positive with both WB and IHC. This one lymphoreticular system (LRS) positive case was an ARQ/VRQ animal, 65 mo of age, derived from our own flock with minimal scrapie-incidence. Further concentration up to 10 mgTE/lane yielded higher signals proportional to the 10× concentration factor, but the diagnostic result in this set of samples was the same as found for the unconcentrated samples.

### Molecular weight and glycoprofiles of PrP^res ^bands

Apparent molecular weights (MW_r_) of the three PrP^res ^bands in WB of RLN digests were compared for a correlation by type of infection (scrapie or BSE), stage of incubation or genotype. A BSE-specific difference in the MW_r _existed and was especially distinct for the aglycosyl moiety, which was about 1kDa larger in scrapie samples than in BSE samples (Table 5). This appeared to be the case for both antibodies 9A2 and L42. Antibody 12B2 (and P4, not shown) only bound well to the scrapie-infected samples (Figure 3) indicating that, as in brain stem tissue [22], a discrimination between scrapie and BSE by dual WB staining is possible using lymphoid tissues, *i.e*. comparing the immunostaining of a sample for PrP-core specific antibody (*e.g*. 9A2 or L42) and PrP^res ^*N*-terminus specific antibodies (12B2 or P4). However, no marked differences in MW_r _for the PrP^res ^bands (not shown) were observed between preclinical and clinical stages of incubation or between scrapie sheep with different genotypes.

**Figure 4:**
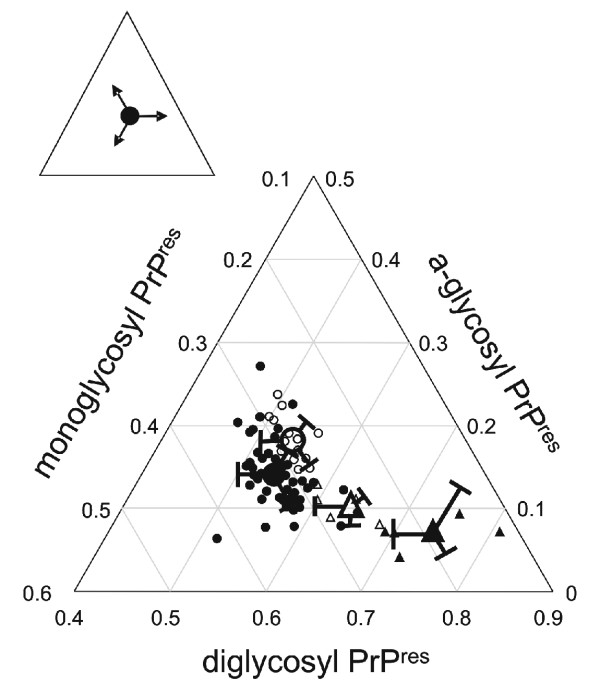
**Glycoprofile of PrP**^res ^**in RLN samples analyzed in scrapie and BSE infected sheep**. Triangular presentation of the proportion of each of three glycoprofile components per individual RLN sample. Symbols correlate with group of sheep: ● – field cases, ◯ – scrapie cases in preclinical study (excluding 2 clinical cases), ▲ – sheep with experimental BSE, △ – sheep in preclinical stage of experimental BSE. The average and SDs per group is indicated by respectively the larger size symbols and bars. The small triangle diagram indicates in which direction from the axes each point should be read.

Glycoprofiles found in individual RLN samples are presented on a triangular graph which allows visualisation of the relative concentration of all three PrP^res ^bands, i.e., the di-, mono-, and aglycosyl moieties (Figure 4). The apparent variations in glycoprofile were further analyzed for correlation with TSE-type, TSE-incubation stage, and genotype. Firstly, for the TSE-types, the diglycosyl fraction of PrP^res ^in scrapie samples of the surveillance group and preclinical study were around 54% (range 45–65), while in BSE-infected sheep in the preclinical and clinical phases, respectively, it constituted 64% (range 58–68) and 74% (range 69–82). These 3 values were significantly different from each other (P < 0.001), and similarly, the aglycosyl and monoglycosyl fractions were statistically different between 4 groups of cases – preclinical scrapie, surveillance, preclinical BSE and clinical BSE (P < 0.001). Secondly, linear regression analyses on both BSE and preclinical scrapie samples yielded slightly decreasing trends with stage of incubation for the aglycosyl and monoglycosyl PrP^res ^fractions, and a concomittant increase in the diglycosyl fraction. The slopes for these stage-dependent trends, however, were unlikely to deviate significantly from zero for either the BSE cases or the cases in the preclinical scrapie study (P > 0.05). This trend was not due to a technical artifact since no correlation between glycoprofile and concentration of PrP^res ^existed. No significant differences were found for the relative concentration of each of the glycoforms of PrP^res ^between genotypes in the scrapie surveillance group (P > 0.05).

## Discussion

This study shows that medial retropharyngeal lymph nodes (RLN) are a suitable target for monitoring scrapie and BSE in sheep, including young lambs. The animals examined were sheep with natural scrapie or experimental BSE in clinical and preclinical stages of the disease, and sheep from an active surveillance program with IHC positive brain but unknown age or clinical status. The diagnostic technique used was Western blotting with several high affinity PrP-specific antibodies. The best results were obtained with antibodies that bind to both scrapie and BSE PrP^res ^(9A2, L42). Sheep of the VRQ/VRQ genotype scored positive from the age of three months, at a stage when the brain was still negative. To intercept as many infected sheep as possible, both brain tissue and lymph nodes have to be examined, since VRQ animals with allele combinations other than VRQ/ARR, and many of the infected ARQ/ARQ sheep, will be positive in RLN much earlier than in brain, whereas a fraction of the ARQ/ARQ, and most ARR/VRQ sheep, may only be positive in the brain. Inclusion in the procedure of a simple concentration step with high PrP^res ^recovery improved the sensitivity of detection of BSE infected animals.

There is still too little knowledge available about the time of appearance of disease-associated PrP in the LRS of sheep with scrapie. This stage will vary with infection pressure, age, strain and genotype. For example, the earliest stage in this study, as in some other studies [16,17], was 2–3 months of age in the most susceptible sheep (genotype VRQ/VRQ), in heavily infected flocks. LRS positivity at preclinical stages of natural scrapie in sheep with other genotypes, such as ARQ/VRQ and ARH/VRQ was noticed at ages varying between 10–24 months, while clinical disease developed later, at more than twice that age [32]. Preclinical accumulation of PrP in LRS tissues has been found in naturally infected Suffolk sheep with ARQ/ARQ genotype between 8–20 months of age [33,34]. Detection of BSE PrP^res ^by WB in RLN of ARQ/ARQ sheep was possible at 6 months post-infection (pmi), the earliest stage investigated, and in all other animals at later stages. The diagnostic score by WB in our BSE-study on RLN was nearly similar as to that obtained by IHC, except for the animal with shortest incubation (6 mpi, Table 3) which by WB was positive but by IHC was negative. However, in this same animal tonsil and ileal Peyer's patches were weakly positive. Discrepancies in the detection of disease-associated PrP in RLN between IHC and WB might be due to sensitivity of tests or a difference in level of detectable disease-associated PrP between left and right side in the head at early stage of appearance.

Signs of preclinical infection have been reported in visceral LRS of ARQ/ARQ Suffolk sheep from 4 months post-infection [35]. It must be borne in mind that the dose of 5 g brain tissue for oral BSEinfection is unusually high and that only ARQ/ARQ animals have been investigated. It is unlikely that such levels have been reached in the field with BSE- contaminated with BSE. However, it is likely, based on IHC studies, that the phenotype of BSE PrP^Sc ^in sheep is not altered by either PrP-genotype or route of challenge, while on the other hand, it might be assumed that the accumulation of PrP^Sc ^varies depending on incoming strain and host genotype [36,37].

Scrapie in sheep is not always accompanied by detectable lymphoid involvement [13,15,16,25,26,32,38,39]. For instance, ARR/VRQ sheep are usually LRS negative. Our data obtained from RLN agree with these observations, with 84% (52/62) of RLN samples in scrapie positive sheep from surveillance scoring positive by WB. Others report a rate between 88–93% for LRS diagnosis in brain positive animals with only XXQ/XXQ genotypes (X's indicate any known variation at respectively codon 136 and 154) [38,40]. In general, scrapie sheep of all PrP genotypes except ARR/VRQ tend to become positive in LRS, while the ARR/VRQs become solely brain positive, and then in only a limited number of animals, at high ages [13,16,17,32-34,40-44]. There are, however, exceptions to the involvement of the LRS with scrapie, as others have reported, and as was also observed in this study [23,40,43,45,46]. One out of our 7 ARQ/ARQ sheep with a scrapie diagnosis in the brain was negative in the lymphoid system. Further, we found all brain positive VRQ/VRQ, VRQ/ARH and ARQ/VRQ sheep to be RLN positive, but Jeffrey [45] reported two (out of 24) ARQ/VRQ sheep to be negative outside the central nervous system. The first ARR/VRQ sheep with involvement of the lymphoid tissue was reported by Ersdal [46] who described an 86 day old lamb with IHC positive Peyer's patches in the ileum. We found two sheep of this genotype with positive lymphoid tissues. Recently, 3 cases of susceptible XXQ/XXQ genotype were reported with positive diagnosis in brain, while negative in all gut-associated lymphoid tissues studied, and which scored positive in the spleen [40]. As well, in infections with the recently described Nor98 strain of scrapie, no PrP^res ^deposits have been detected so far in lymphoid tissues. The Nor98 condition has only been diagnosed by examination of the brain, and then, primarily the cerebellum regions [47]. Likewise, no deposits of PrP^Sc ^have been detected in LRS tissues of sheep intracerebrally infected with CH1641, an experimental sheep scrapie strain that exhibits similar migrational properties for PrP^res ^as BSE in sheep [21,48] (our own observations). Other techniques, such as ELISA and IHC with brain or LRS, might assist in further classification of dubious cases [48,49]. In summary, while the involvement of peripheral tissues in all clinical or preclinical classical scrapie is not absolute, nevertheless, it occurs with sufficient regularity to be of use in surveillance systems and potentially provides earlier pre-clinical diagnosis than would be achieved by analysis of CNS tissues.

Different methods for PrP^res ^concentration from homogenates were used, depending on the tissue source: centrifugation for RLN without additives, and centrifugation after addition of alcohols, for brain tissue. By allowing 20 mg tissue equivalents to be tested, both methods lead to increased sensitivity of detection of PrP^res ^by WB compared to no centrifugation. For RLN, it improved BSE detection. However, four scrapie samples, that were weakly positive by IHC, were not detected even after concentration: 2 preclinical RLN samples from VRQ/VRQ sheep with scrapie at two months of age, and 2 RLN samples from routine diagnosis – 1 ARQ/ARQ and 1 ARR/VRQ sheep. IHC of these few non-corresponding samples appeared to be only weakly positive, indicating that, with WB, it is possible, in principle, to detect most cases identified by IHC. In brain samples from animals between 10–17 months of age (Table 2), WB might have missed positive diagnosis in 4 preclinical cases, since these analyses possibly obex parts lacking dorsal motor nucleus of the nervus vagus where the first positivity can be found [25]. In our hands the PrP^Sc ^precipitation technique described by Wadsworth [24], which is based on the presence of phophotungstic acid, gave unsatisfactory recoveries (≤ 60%) after PK digestion. Recoveries were much better if precipitation preceded PK digestion, but this approach changed the mobility of the PrP^res ^bands, making it difficult to rely on molecular weight determination to distinguish between strains.

When analysed by WB, glycoprofiles and apparent molecular weights of PrP^res ^in RLN digests were similar to those reported for brain tissue of scrapie or BSE infected sheep [22]. This means firstly, that, using RLN, both TSEs can be detected in a single screening with the PrP-core specific antibodies L42 or 9A2. Secondly, differentiation of BSE and scrapie can be done subsequently, in a dual antibody test using two blots, one with a PrP-core specific antibody, such as L42 or 9A2, and the other with a PrP^res ^*N*-terminus specific antibody, such as P4 or 12B2. All these antibodies have high affinity for ovine PrP. Glycoprofile analyses can further support the differential diagnosis of scrapie and BSE, where the relative amount of the diglycosyl moiety of PrP^res ^is lower in scrapie than BSE. However, glycoprofiling remains an inconclusive tool in discriminating scrapie from BSE cases since there is overlap in glycoprofile between the scrapie and BSE cases (Figure 4). In other studies glycoprofile analyses in sheep have yielded too divergent results in scrapie cases to allow a discriminatory diagnosis between scrapie and BSE [18,20,50,51]. Nevertheless, the relatively high concentration of the diglycosyl PrP^res ^fraction in BSE-infected animal species, including humans with a variant form of Creutzfeldt-Jakob disease, remains noteworthy [21,52-54]. A new factor in glycoform analyses of PrP^res ^is the possible dependence on stage of incubation. Though this was not statistically significant, it was striking that in both our sheep TSE incubation studies, experimental BSE in ARQ/ARQ sheep and natural scrapie in VRQ/VRQ animals, a correlation was found, revealing an age dependent increase in the diglycosyl moiety of PrP^res ^with a concomittant decrease in the a- and monoglycosyl fractions. This association was not due to a technical artifact since no correlation between glycoprofile and concentration of PrP^res^. These time-related variations in protein glycosylation could be due to disease status, but normal age dependent variations in posttranslational processes during protein synthesis should also be considered [55]. Finally, these glycoprofile variations further indicate that, as a tool for discriminating scrapie from BSE, glycoprofiling in sheep is a rather unreliable parameter when the age of the animal and the strain properties of the isolate are not known. Likewise, in brain, further studies are needed to establish a time-dependent relation of PrP^res^-glycosylation.

This study was performed on samples collected during the years 2002–2003 and appears to be a rather representative cross-section of the Dutch sheep population, with respect to the presence of the different genotypes (compare for the years 1999–2001 in [22]). It remains to be seen how consistently the RLNs score in different age groups, genotypes and preclinical stages. Nevertheless, the efficacy of detecting scrapie and BSE infection in current monitoring programs can be highly improved for animals in a stage where the TSE agent has not yet invaded the central nervous system, i.e., at ages younger than 18 months. At older ages, both brain and RLN testing is needed to optimally assure absence of TSE. It is expected that lymphoid testing also enhance surveillance efficiency in goats, where the lymphoid system usually is involved [11,14,56] (Van Keulen personal communication).

## Conclusion

This study demonstrates that Western blotting can be used for routine screening of classical scrapie and BSE in retro-pharyngeal lymph nodes of sheep at slaughter with a sensitivity nearly the same as immunohistochemistry. A concentration step for PrP^res ^is required. The difference in polypeptide length of PrP^res ^between BSE and scrapie can be unequivocally confirmed by dual antibody staining using two classes of antibodies: one which binds to the N-terminus of PrP^res ^in scrapie only, and one which binds to the core of PrP^res ^in both BSE and scrapie.

## Methods

### Sheep and tissues

Sheep used were from our own flock, from slaughter or fallen stock. In total, 103 sheep were used in this study.

For preclinical and clinical scrapie, 24 animals with known ages were used (Table 2). These animals have been described previously in immunohistochemical (IHC) pathogenesis studies [25,26] and were bred within our own Texelcross flock with natural scrapie. The animals were euthanized and sampled at ages from 2-26 months. 18 sheep were of VRQ/VRQ genotype. Potentially negative controls from the same flock comprised 4 ARR/VRQ (ages 17, 17, 24, and 24 months) and 2 ARR/ARR animals (ages 3 and 6 months).

For preclinical and clinical BSE, 11 sheep of ARQ/ARQ genotype were orally fed with bovine BSE brain as previously described [27]. Four of these animals were kept until clinical signs of disease appeared, the others were euthanized between 6 – 19 months post-infection. These animals were positive by IHC for PrP^Sc ^in their tonsils at biopsy or at autopsy (Table 3).

The remaining 68 sheep were from different surveillance sources, most of them of unknown ages (*see for individual data Table in *"additional file 1"). Within this group, 12 animals with and without clinical suspicion of scrapie were obtained from either our own flock with natural scrapie (n = 5), our own flock maintained with minimal scrapie-incidence (n = 3), private farms with clinically suspect cases (n = 3), or a private farm with known history of scrapie (n = 1, normal animal). Of the remaining sheep, most were diagnosed through the active surveillance program for slaughter (n = 50) and fallen stock (n = 6). The heads of the animals in the monitoring program had been kept at 4°C for up to three days. Seven of the 68 animals were TSE negative in the brain by rapid testing and IHC, with one of these 7 being TSE positive in tonsil and RLN by IHC.

Brain and lymphoid tissues were collected for IHC and biochemical assays. From brain, one parasagitally cut longitudinal half was fixed in formaldehyde and embedded for pathological and immunohistochemical analysis [25]; the other smaller part was stored at -20°C. Lymphoid organs from one side of the animal were sampled for IHC, and from the other side were stored at -20°C for biochemical assays.

### Scrapie diagnosis

The Prionics-Check Western blot method for active monitoring of BSE in cattle was used [57] for routine scrapie diagnosis on brain stem at the obex region. This method uses digestion with proteinaseK at 50°C and PrP-specific monoclonal antibody 6H4 for detection. Scrapie was further confirmed by immunohistochemical (IHC) analysis on brain and lymphoid tissues according to established procedures with the various antibodies described and characterized previously [13,27,58].

### Genotyping

Blood from sheep were used for PrP genotyping for PrP codons 136, 154, and 171 using two genotyping techniques, i.e., TaqMan analysis and, for confirmation of Arg, Gln or His at codon 171, Pyrosequencing [22,44]. To exclude mistakes by exchange of samples, genotype was also checked in homogenates of RLN and brain stem.

### Antibodies and fine epitope mapping by Pepscan analysis, peptide synthesis and blocking ELISA

Antibodies used for WB were from different sources. The following murine PrP-specific monoclonal antibodies (mAbs) were purchased: P4, L42 (R-Biopharm, Almere, The Netherlands), 8G8, SAF70, SAF84 (SPI-BIO, Montigny le Bretonneux, France), 34C9 and 6H4 (Prionics AG, Zurich, Switzerland). These mAbs were prepared as described [59-62]. MAb 94B4 was previously described [22] and mAbs 12B2 and 9A2 were newly prepared using PrP-knockout mice [63] immunized with peptide GGGGWGQGGTHGQWNKPSK (bovine PrP 97–115), conjugated through a cysteine at its Cterminus to Keyhole limpet hemocyanine using previously described procedures [64]. Immunisations were carried out in the presence of incomplete Freund's adjuvant (first injection) or adjuvant CoVaccine HT (CoVaccine, Utrecht, The Netherlands) in 4 administrations evenly distributed over a period of 63 days. Animals were bled and spleens used for hybridisation to Sp2/0 myeloma cells according to standard procedures. Screening for PrP-specific antibodies in sera and culture supernates was performed by indirect ELISAs using the peptide, recombinant ovine PrP (kind gift of T. Sklaviadis, Aristotle University, Thessaloniki, Greece) and bovine PrP (Prionics AG) as coated antigens at 0.2, 0.2, and 0.1μg/ml, respectively. The fine epitope specificities of 12B2, 9A2 and L42 were determined by Pepscan analyses with overlapping 15-mer solid phase peptides using the ovine PrP sequence as sequence basis as previously described [22,65], and further confirmed in ELISA by blocking antibody binding to coated recombinant ovine PrP, using synthetic peptides. Blocking ELISA was performed as follows: polystyrene microtiter plates were coated overnight at 4°C with recombinant ovine PrP at 0.1 μg/ml in 6 M guanidinium-HCl in PBS (138 mMNaCl, 2.7 mM KCl, 8 mM Na_2_HPO_4_, 2.8 mM KH_2_PO_4_, pH7.2). Plates were washed with 0.05% Tween-20 in water. In separate microtiter plates antibody plus peptide was preincubated overnight in 1% (w/v) Tween80, 4% (v/v) horse serum, 0.35 M NaCl in PBS (ELISA medium). The antibody-peptide mixture was transferred to the plate coated with PrP. After 1 h at ambient temperature, plates were washed as before and further developed by addition of horseradish peroxidase-rabbit anti-mouse Ig conjugate (DAKO, Denmark) diluted 1/1000 in ELISA medium. Bound antibody was spectrophotometrically measured at 450 nm after addition of 3,3',5, 5'tetramethylbenzidine for 20 min and stop of the reaction with sulphuric acid. Synthetic peptides used for blocking in solution were: ovine PrP145-177 and PrP89-107 (Genbank accession number AJ567985).

### Tissue treatment

Tonsils and medial retropharyngeal lymph node tissue were macroscopically freed from surrounding fat and connective tissue. 10% homogenates were prepared by homogenisation in lysis buffer consisting of 0.5%Triton X-100, 0.5%Nadeoxycholate, in PBS. Homogenisation was performed for 1 min in either 50 ml Falcon tubes and OmniTP equipment with disposable probes (Omni International Inc., Warrenton, VA, USA) at 30,000 rpm or for 45 s at 23,000rpm in Prypcon Lymph Node 80300/B vials with MediFASTH homogeniser (Consul AR SA, Villeneuve, Switzerland). For storage purposes, homogenates were clarified in 1.5 ml Eppendorf vials by centrifugation at 10,000 × g for 10 min at ambient temperature. After addition of 10 μl of 550 μg/ml proteinaseK (PK, 30 U/mg, 124568, Merck, Darmstadt, Germany) in PBS to 100 μl of homogenate, digestion was performed for 40 min at 50°C. The reaction was subsequently by addition of 10μl of a Pefabloc solution (3 mg/ml Pefabloc SC in PBS; Roche, Almere, The Netherlands), 100 μl 2× sample buffer (20% [w/v] sucrose, 0.282M Tris-Base, 0.212M Tris-HCl, 4% [w/v] Nadodecylsulphate, 1.0 mM EDTA, 0.038% [w/v] bromephenol blue, 4% [v/v] β-mercaptoethanol), and heating for 5 min at 95°C. On some occasions, concentration of PrP^res ^in RLN was first carried out by centrifugation of 100 μl digest in 1.5 ml vials at 14,000 rpm, 21,000 xg for 1 h in an Eppendorf 5417R centrifuge at 4°C as recently described [66]; pellets were dissolved by subsequent addition of 10 μl 0.1 % N-lauroylsarkosine in PBS and 10 μl of 2× sample buffer. Samples were heated for 5 min at 95°C.

Brain stem tissue from scrapie sheep was homogenized and digested either according to the protocol of a routine test (PrionicsCheck) or treated as above for RLN. To enhance sensitivity, PrP^res ^was concentrated as follows: after digestion with PK and addition of Pefabloc, the digest was mixed with 100 μl of a mixture of propan-2-ol/n-butan-1-ol (1/1, v/v) and centrifuged for 5 min at 21,000 xg in a microcentrifuge at room temperature (Eppendorf). The pellet was finally dissolved in 1× sample buffer (2 × sample buffer diluted with an equal volume of water). The recovery of PrP^res ^in this procedure for concentration was consistently >90% when comparing equal tissue equivalents of unconcentrated and concentrated material, allowing the application of 20 mg tissue equivalents (TE) per lane, instead of 1 mg TE in unconcentrated state. This concentration method employed the protein precipitating properties of alcohols while removing SDSPAGE-disturbing components from brain tissue.

### Deglycosylation treatment with PNGaseF

Removal of asparagine-linked oligosaccharides after PK digestion was performed as follows, After blocking the PK reaction with Pefabloc, 10 μl of denaturation buffer (5% sodium dodecyl sulfate [SDS], 10% β-mercaptoethanol in 20 mM Tris-HCl, 150 mM NaCl, 2 mM EDTA [pH 7.5]) was added to the sample. This was heated for 10 min at 95°C. After cooling, 10 U of PNGaseF (New England Biolabs, Beverley, USA) was added and incubated overnight at 37°C. To stop the reaction, 100 μl of 2× sample buffer was added and the mixture heated for 5 min at 95°C.

### SDS-PAGE, Western blotting, and immunochemical development

SDS-PAGE was performed with 10-well precast 1 mm 12% BisTris NuPAGE gels (NuPAGE gel electrophoresis system with MOPS buffer; Invitrogen, Breda, The Netherlands). Molecular weight markers used were MagicMark and SeeBlue Mark12 (Invitrogen). Sample volumes applied varied between 10 to 20 μl per lane, or 0.5 – 10 mg tissue equivalents (TE)/lane. Electrotransfer onto polyvinylidene difluoride membranes (PVDF, Immobilon-P; Millipore, Bedford, Mass.) and immunostaining were performed according to established procedures [67, 68]. After electrotransfer, blots were blocked for 30 min with 5%skim milk protein in antibody incubation solution (25 mM Tris-HCl, 0.15 M NaCl, 2.7 mM KCl, 0.05% Tween20 at pH7.4). Primary antibodies were used at concentrations between 0.2–2 μg IgG/ml in antibody incubation solution. Secondary antibody used was rabbit anti-mouse immunoglobulinG conjugated to alkaline phosphatase (Dako, Glostrup, Denmark). Signal was developed with CDPStar by following the supplier's instructions (Tropix, Bedford, Mass.) and were recorded on photographic film, usually with exposure times between 1–45 min (Hyperfilm ECL; Amersham, Buckinghamshire, United Kingdom). Molecular weights were determined according to a method described [69]. To estimate glycoprofiles of PrP^res ^i.e. the relative proportions of di-, mono-, and aglycosyl fraction, films were recorded with an Agfa Duoscan T200XL scanner and further processed with GelPro software (MediaCybernetics, Silver Spring, MD) from which calculation of mutual densities of the three protein bands was possible. In experiments to compare the relative affinity for ovine PrP^res^, antibodies were applied in concentration series on PVDFstrips from blots transferred from single well gels run with ovine scrapie infected brain stem homogenates varying between 1.25–20 mg tissue equivalents (TE).

### Statistical analyses

One-way analyses of variance (ANOVA) were carried out to establish whether variations between groups of data, *in casu *glycoform fractions of PrP^res^, were greater than expected; if so, subsequent differences between pairs of groups were considered significant if the probability of a difference was <0.05 in multiple-comparisons tests according to the Student-Newman-Keuls test. Linear regression analyses were performed for an increasing or decreasing trend with disease incubation on data obtained in preclinical scrapie and experimental BSE infection study using a P value of <5% as confidence interval for concluding that the data are unlikely to be sampled from a population in which the slope is zero. The linearity of these curves could not be reliably established due to the small number of samples. The software used for these calculations was Instat Biostatistics from Graph-Pad Software, San Diego, CA.

## Authors' contributions

JPML supervised this study, evaluated all experimental aspects, and wrote the final version of the manuscript; JGJ designed crucial steps of the whole work and performed nearly all practical activities; JHFE contributed to concentration experiments in RLNs; AB contributed the genotyping studies; FGvZ contributed to tissue sampling and intellectual decisions; LJMvK contributed by IHC, tissue sampling and animal experiments.

## Supplementary Material

Additional File 1 A table with data from individual sheep to supplement summarized results of table 4. Sheep from surveillance with mostly unknown age at death. Table heading: **Sheep from surveillance**. Click here for file

## Figures and Tables

**Table 1:** Epitope specificities of 4 mouse monoclonal antibodies used.

antibody	elicited with sequence	epitope core by Pepscan analysis^†^	blocking ELISA^‡^	
			89–107	145–177
12B2	bovine PrP97-115^§^	93WGQGG97	yes	no
P4	ovine PrP89-104	93WGQGGSH99	yes	no
9A2	bovine PrP97-115^§^	102WNK104	yes	no
L42	ovine PrP145-163	148YEDRYY153	no	yes

†Mapping of antigenic sites in the ovine PrP sequence for 4 monoclonal antibodies by Pepscan analysis.

Shown are shared sequences (core of epitope) of overlapping 15-mer antigenic peptides for antibodies 12B2, P4, 9A2 and L42 from Figure 1. Numbers refer to ovine PrP sequence Genbank accession nr. AJ567985.

^‡^Blocking was 100% in all positive cases and 0% in all negative cases with peptide concentrations of ≥ 0.5 μg/ml and antibody concentrations ≤ 0.5 μg/ml.

^§^Corresponds to ovine PrP sequence 89–107. Antibodies produced by [60]

**Table 2:** Sheep with preclinical scrapie*.

			diagnosis			
			
sheep nr	genotype	age (m)	IHC		WB	
			
			brain	RLN^†^	brain^‡^	RLN^‡^
PS-5	VRQ/VRQ (n = 18)	2	-	1+	-	-
PS-6		2	-	1+	-	-
PS-3		3	-	2+	-	2+
PS-4		3	-	-	-	-
PS-1		4	-	4+	-	3+
PS-12		4	-	3+	-	2+
PS-7		5	-	4+	-	3+
PS-8		5	-	4+	-	1+
PS-10		5	-	4+	-	1+
PS-11		5	-	3+	-	2+
PS-18		10	1+^§^	4+	-	2+
PS-17		13	1+	4+	-	2+
PS-15		14	2+	4+	-	2+
PS-16		14	2+	4+	-	3+
PS-14		17	3+	4+	1+	2+
PS-13		21	3+	4+	3+	2+
PS-23		26^¶^	3+	4+	3+	2+
PS-24		26^¶^	3+	4+	3+	1+
PS-21	ARR/VRQ (n = 4)	17	-	-	-	-
PS-22		17	-	-	-	-
PS-19		24	-	-	-	-
PS-20		24	-	-	-	-
PS-2	ARR/ARR (n = 2)	3	-	-	-	-
PS-9		6	-	-	-	-

* open flock of Texel cross breed with natural scrapie; – means no PrP or PrP^res ^detected.

† number of plus signs indicate % of follicles PrP-positive 1+ = <25%, 2+ = 25-50%, 3+ = 50–75%, 4+ = 75–100%.

^‡ ^1+ = weak signal for all three PrP^res ^bands; 2+ = PrP^res ^bands well visible; 3+ = PrP^res ^bands so dark that diglycosyl and monoglycosyl are merging together at 6 min film exposure. Results obtained with either of the antibodies 12B2, P4 or 9A2 after SDS-PAGE of non-concentrated samples, or with 12B2 and concentrated samples.

^§ ^In a previous study, another 10 m old RLN positive animal was found negative in the brain by IHC [25].

^¶ ^Clinical signs.

**Table 3:** Post mortem diagnosis in RLN of sheep with experimental BSE in preclinical and clinical status*.

sheep nr.	m.p.i. at euthanasia	IHC^†^	WB^‡^
2117	6	-^¶^	2+
6869	9	+	1+
2114	12	+	2+
3903	13	+	2+
3907	15	+	2+
3905	17	+	2+
2112	19	+	2+
2140	21^§^	+	2+
2115	21^§^	+	2+
2138	23^§^	+	2+
2116	24^§^	+	2+

* Oral inoculations occurred between 4–6 mo of age. Each animal received 5 g of bovine BSEinfected brain by oral route [27].

^† ^Positive in tonsil and RLN.

^‡ ^1+, weak signal for all three PrP^res ^bands. 2+ PrP^res ^bands clearly visible. Results after concentration obtained with either of the antibodies 9A2 or L42; antibody 12B2 scored negative in all samples.

^§^Clinical signs.

^¶ ^Weakly positive in tonsil and ileal Peyer's patches.

**Table 4:** Scrapie diagnosis in medial retropharyngeal lymph nodes from animals mostly with unknown age at death*.

	scrapie positive in CNS		scrapie negative in CNS	
	WB	IHC	WB	IHC
*all genotypes*	51/61	53/61	1/7	1/7
				
*per genotype*				
ARQ/ARQ	5/7	6/7	0/1	0/1
ARQ/ARR	none	none	0/2	0/2
VRQ/VRQ	6/6	6/6	none	none
ARQ/VRQ	32/32	32/32	1^†^/2	1^†^/2
ARH/VRQ	7/7	7/7	none	none
ARR/VRQ^‡^	1/9	2/9	0/2	0/2

* Sources of animals were own flocks, clinical suspects, and slaughtered or fallen stock animals aging >18 m collected under EU surveillance program for small ruminants (see Methods section *and for individual data Table in *"additional file 1"). Figures indicate the number of positives/total number of animals analyzed. The analyses on central nervous system (CNS) were performed by Prionics Check and IHC, and concerned obex tissue analysis.

^† ^Animal aging 65 m, found dead from minimal scrapieincidence pressure flock.

^‡ ^There were two samples (RLN65 and RLN67) which in three WB tests yielded once a weakly positive result, and twice (including after a concentration treatment) a negative result. In IHC, RLN65 was weakly positive, RLN 67 negative.

**Table 5:** Molecular weight estimations of PrP^res ^form in retropharyngeal lymph nodes and brain stem*.

	**scrapie**			
	RLN			brain stem
		
antibody	L42	9A2	12B2^†^	9A2
				
*PrP^res^form*				
diglycosyl	28.8 ± 0.7	28.9 ± 1.9	29.2 ± 0.8	28.6 ± 1.4
monoglycosyl	22.4 ± 0.5	22.9 ± 1.0	22.8 ± 0.2	22.7 ± 0.5
aglycosyl	18.8 ± 0.4	19.0 ± 0.8	18.9 ± 0.1	18.6 ± 0.5
	**BSE**			

	RLN			brain stem
		
antibody	L42	9A2	12B2^†^	9A2
				
*PrP*^res^*form*				
diglycosyl	28.5 ± 1.3	28.9 ± 2.0	N.R.	28.3
monoglycosyl	21.9 ± 1.1	22.2 ± 2.1	N.R.	22.5
aglycosyl	17.8 ± 0.8	18.0 ± 1.7	N.R.	18.0

* Values are in kDa and presented as average ± SD, from 3 different experiments with at least 4 different individual animals per experiment, except for mAbs 12B2 (n = 2) and 9A2 on BSE infected brain stem, n = 1.

^† ^Antibody 12B2 yielded the same MW_r _as P4 in scrapie samples; N.R. = no reactivity.
