# Supplementary material for: Rapid and discriminatory diagnosis of scrapie and BSE in retro-pharyngeal lymph nodes of sheep
Source: BMC Vet Res. 2006 Jun 9;2:19. doi: 10.1186/1746-6148-2-19 (PMC1544330; doi:10.1186/1746-6148-2-19)
Supplement: Additional File 1 — A table with data from individual sheep to supplement summarized results of table 4. Sheep from surveillance with mostly unknown age at death. Table heading: Sheep from surveillance. [file 1746-6148-2-19-S1.doc]

**Table with data on individual sheep**

**Sheep from surveillance.**

| sheep code | origin^a^ | genotype  (obex+head) | clinical status at collection (age m) | diagnosis | | | |
| --- | --- | --- | --- | --- | --- | --- | --- |
|  |  |  |  | IHC | | WB | |
|  |  |  |  | brain | tonsil^b^ | brain^c^ | RLN^d^ |
| RLN01 | farm Y | ARH/VRQ | clin. susp | + | + | ++ | + |
| RLN02 | farm Y |  | clin. susp | + | + | ++ | ++ |
| RLN22 | S |  | N.A. | + | + | + | ++ |
| RLN24 | S |  | N.A. | + | + | + | +++ |
| RLN26 | S |  | N.A. | + | + | +++ | +++ |
| RLN31 | S |  | N.A. | + | + | +++ | ++ |
| RLN33 | S |  | NS | + | + | +++ | ++ |
| RLN03 | OF | ARQ/ARQ | found dead (47) | + | + | ++ | + |
| RLN12 | OFM |  | clin. neg (62) | - | - | - | - |
| RLN14 | OF |  | N.A. | + | + | +++ | ++ |
| RLN16 | S |  | N.A. | + | + | ++ | ++ |
| RLN17 | S |  | N.A. | + | + | + | ++ |
| RLN42 | S |  | N.A. | + | +/- | +++ | - |
| RLN64 | S |  | N.A. | + | - | + | - |
| RLN68 | S |  | N.A. | + | + | + | ++ |
| RLN06 | OF | ARQ/ARR | found dead (134) | - | - | - | - |
| RLN09 | OF |  | clin. neg (136) | - | - | - | - |
| RLN04 | OFM | ARQ/VRQ | found dead (84) | - | - | - | - |
| RLN11 | OFM |  | clin neg (85) | - | + | - | + |
| RLN13 | S |  | N.A. | + | + | ++ | ++ |
| RLN18 | S |  | N.A. | + | + | ++ | ++ |
| RLN21 | S |  | N.A. | + | + | +++ | ++ |
| RLN23 | S |  | N.A. | + | + | ++ | ++ |
| RLN25 | S |  | N.A. | + | + | ++ | ++ |
| RLN27 | S |  | N.A. | + | + | + | +++ |
| RLN28 | S |  | N.A. | + | + | ++ | +++ |
| RLN30 | S |  | N.A. | + | + | +++ | +++ |
| RLN32 | S |  | N.A. | + | + | ++ | ++ |
| RLN35 | OF |  | N.A. | + | + | +++ | +++ |
| RLN36 | FS |  | N.A. | + | + | ++ | ++ |
| RLN39 | S |  | N.A. | + | + | +++ | ++ |
| RLN40 | S |  | N.A. | + | + | +++ | ++ |
| RLN41 | FS |  | N.A. | + | + | +++ | ++ |
| RLN43 | S |  | N.A. | + | + | +++ | ++ |
| RLN44 | S |  | N.A. | + | + | +++ | +++ |
| RLN47 | S |  | N.A. | + | + | +++ | + |
| RLN48 | S |  | N.A. | + | + | +++ | ++ |
| RLN49 | S |  | N.A. | + | + | +++ | ++ |
| RLN50 | S |  | N.A. | + | + | +++ | ++ |
| RLN52 | S |  | N.A. | + | + | +++ | ++ |
| RLN54 | S |  | N.A. | + | + | +++ | ++ |
| RLN55 | S |  | N.A. | + | + | +++ | ++ |
| RLN56 | S |  | N.A. | + | + | +++ | ++ |
| RLN57 | S |  | N.A. | + | + | +++ | ++ |
| RLN58 | S |  | N.A. | + | + | +++ | ++ |
| RLN59 | S |  | N.A. | + | + | +++ | ++ |
| RLN60 | S |  | N.A. | + | + | +++ | ++ |
| RLN61 | FS | ARQ/VRQ | N.A. | + | + | +++ | ++ |
| RLN63 | S |  | N.A. | + | + | ++ | ++ |
| RLN69 | S |  | N.A. | + | + | ++ | +++ |
| RLN70 | S |  | N.A. | + | + | ++ | ++ |
| RLN08 | OF | ARR/VRQ | found dead (49) | - | - | - | - |
| RLN10 | farm M |  | clin. neg. | - | - | - | - |
| RLN15 | S |  | N.A. | + | +/- | + | ++ |
| RLN19 | S |  | N.A. | + | - | + | - |
| RLN20 | S |  | N.A. | + | - | + | - |
| RLN34 | S |  | N.A. | + | - | + | - |
| RLN37 | S |  | N.A. | + | - | ++ | - |
| RLN62 | FS |  | N.A. | + | - | + | - |
| RLN65 | S |  | N.A. | + | +/- | ++ | +/-^e^ |
| RLN66 | S |  | N.A. | + | - | ++ | - |
| RLN67 | S |  | N.A. | + | - | ++ | +/-^e^ |
| RLN05 | farm O | VRQ/VRQ | clin. susp | + | + | N/A | ++ |
| RLN07 | OF |  | found dead (23) | + | + | ++ | + |
| RLN38 | S |  | N.A. | + | + | + | +++ |
| RLN46 | S |  | N.A. | + | + | +++ | ++ |
| RLN51 | S |  | N.A. | + | + | ++ | +++ |
| RLN53 | S |  | N.A. | + | + | +++ | ++ |

These 68 sheep were from different surveillance sources, most of them at unknown ages. Within this group, 12 animals with and without clinical suspicion of scrapie were obtained from either own flock with natural scrapie (n=5, RLN3, 6, 7, 8, and 9), own flock maintained with minimal scrapie-incidence (n=3, RLN4, 11, and 12), private farms because of clinical suspicion (n=3, RLN1, 2 and 5), or private farm with known history of scrapie (n=1, RLN10). Of the remaining sheep most were diagnosed through the active surveillance program for slaughter (n=50) and fallen stock (n=6, RLN14, 35, 36, 41, 62, and 69). The heads of the animals in the monitoring program had been kept at 4^o^C for up to three days.

^a^ S=slaughter, FS=fallen stock, OF=own scrapie flock, OFM=own flock with minimal scrapie pressure, farms Y, M and O = farms with scrapie history; NA=no data available.

^b^ IHC: +/- = weakly positive and in less than 25% of follicles.

^c^ Prionics Check Western blot: + = 1 or 2 PrP^res^ bands visible, ++ = three bands visible, +++ = 3 bands fusing together by overexposure. N/A: not available.

^d^ WB RLN: +/- = samples that scored one in three tests weakly positive, + = positive at film exposure ≥15 min, ++ = positive at all exposure conditions; +++ = strongly positive with bands fusing. Antibodies used: 12B2, 9A2 and P4.

^e^ Samples which after three tests yielded once a weakly positive result, and twice (including after a concentration treatment) a negative result.
